# Barrier Enclosure for Endotracheal Intubation in a Simulated COVID-19 Scenario: A Crossover Study

**DOI:** 10.5811/westjem.2020.7.48574

**Published:** 2020-08-17

**Authors:** Torrey A. Laack, Franziska Pollok, Benjamin J. Sandefur, Aidan F. Mullan, Christopher S. Russi, Suraj M. Yalamuri

**Affiliations:** * Mayo Clinic, Department of Emergency Medicine, Rochester, Minnesota; † Mayo Clinic, Mayo Clinic Multidisciplinary Simulation Center, Rochester, Minnesota; ‡ Mayo Clinic, Division of Biomedical Statistics and Informatics, Rochester, Minnesota; § Mayo Clinic, Department of Anesthesiology, Rochester, Minnesota

## Abstract

**Introduction:**

Barrier enclosures have been developed to reduce the risk of COVID-19 transmission to healthcare providers during intubation, but little is known about their impact on procedure performance. We sought to determine whether a barrier enclosure delays time to successful intubation by experienced airway operators.

**Methods:**

We conducted a crossover simulation study at a tertiary academic hospital. Participants watched a four-minute video, practiced one simulated intubation with a barrier enclosure, and then completed one intubation with and one without the barrier enclosure (randomized to determine order). The primary outcome measure was time from placement of the video laryngoscope at the lips to first delivered ventilation. Secondary outcomes were periprocedural complications and participant responses to a post-study survey.

**Results:**

Proceduralists (n = 50) from emergency medicine and anesthesiology had median intubation times of 23.6 seconds with practice barrier enclosure, 20.5 seconds with barrier enclosure, and 16.7 seconds with no barrier. Intubation with barrier enclosure averaged 4.5 seconds longer (95% confidence interval, 2.7–6.4, p < .001) than without, but was less than the predetermined clinical significance threshold of 10 seconds. Three complications occurred, all during the practice intubation. Barrier enclosure made intubation more challenging according to 48%, but 90% indicated they would consider using it in clinical practice.

**Conclusion:**

Experienced airway operators performed intubation using a barrier enclosure with minimal increased time to procedure completion in this uncomplicated airway model. Given potential to reduce droplet spread, use of a barrier enclosure may be an acceptable adjunct to endotracheal intubation for those familiar with its use.

## INTRODUCTION

### Background

On December 31, 2019, the World Health Organization (WHO) was first notified about a cluster of cases of pneumonia in Wuhan City, Hubei Province, China.^1^,^2^ The identified virus, named SARS-CoV-2, causes the disease now termed COVID-19.^2^–^4^ On March 11, 2020, COVID-19 was officially labeled a pandemic.^2^,^5^ The infection continues to spread rapidly, and affects the majority of countries across the globe.^6^

Aerosol-generating procedures (AGP), such as bag-mask ventilation and endotracheal intubation (ETI), are high risk for nosocomial transmission of respiratory infections to healthcare providers.^7^,^8^ COVID-19 is transmitted by contact and droplet transmission, while aerosol spread remains uncertain.^9^ SARS-CoV-2 is stable in aerosol under laboratory conditions, indicating that aerosol transmission is a plausible means of transmission of COVID-19 to healthcare providers.^10^,^11^ Furthermore, SARS-CoV-2 may remain infectious in aerosols for hours.^10^ Transmission from critically ill patients is a significant source of anxiety for healthcare providers,^12^ and early reports suggested 19% of COVID-19 cases were in healthcare providers.^13^

### Importance

Guidelines have emerged to encourage safe care of patients during the COVID-19 pandemic while minimizing risk to healthcare providers.^14^ A central component of the guidelines is the proper use of personal protective equipment (PPE) to decrease nosocomial infection with COVID-19. However, shortages of adequate PPE are widespread.^15^ Novel strategies have been developed to mitigate nosocomial spread during ETI, especially given PPE shortages. Physical barriers to shield the proceduralist’s face from the patient’s airway have been developed.

At the most basic level, a simple box made out of corrugated fiberboard and transparent plastic wrap has been described.^16^ Instructions for an “aerosol box,” which can be made inexpensively out of acrylic or polycarbonate material and is reusable after proper cleaning, are widely available on the Internet.^17^ This original design has since been modified to make it larger and more accommodating to different-sized patients while also better allowing other techniques, such as use of a gum elastic bougie.^18^,^19^ On May 1, 2020, the US Food and Drug Administration issued an Emergency Use Authorization for protective barrier enclosures.^20^

A barrier enclosure device was tested using dye and a simulated cough and was reported to potentially reduce contamination of the proceduralist.^21^ However, the use of barrier enclosure devices is not without criticism. Questions regarding the applicability to larger patients and the limited space in which to work for such a critical procedure as ETI remain unanswered.^22^,^23^ Prior studies have shown minimal impact of PPE use on ETI success,^24^,^25^ but use of a barrier enclosure may lead to breaches in PPE.^19^ Of paramount concern is that use of the barrier enclosures seems to be spreading through social media and the Internet despite little evidence supporting their safety or efficacy.^22^,^23^

### Goals of This Investigation

Before implementing widespread use of a novel device, testing is needed to establish an evidence base supporting its safety. Prior studies have demonstrated that negative patient outcomes are associated with delayed first-pass intubation success.^26^–^28^ The use of a barrier enclosure, especially by individuals who have had little or no prior experience with the device, may delay time to successful intubation or increase periprocedural complications. We sought to determine whether use of a barrier enclosure delays time to successful intubation by experienced airway operators.

**Population Health Research Capsule**

What do we already know about this issue?

Barrier enclosures have been developed to reduce the risk of COVID-19 transmission to healthcare providers during intubation.

What was the research question?

We sought to determine whether a barrier enclosure delays time to successful intubation by experienced airway operators.

What was the major finding of the study?

Experienced clinicians performed intubation using a barrier enclosure with minimal increased time to procedure completion.

How does this improve population health?

Given risk of COVID-19 transmission to healthcare providers during intubation, use of a barrier enclosure may be an acceptable adjunct for those familiar with its use.

## METHODS

### Study Design and Setting

**Figure 1:**
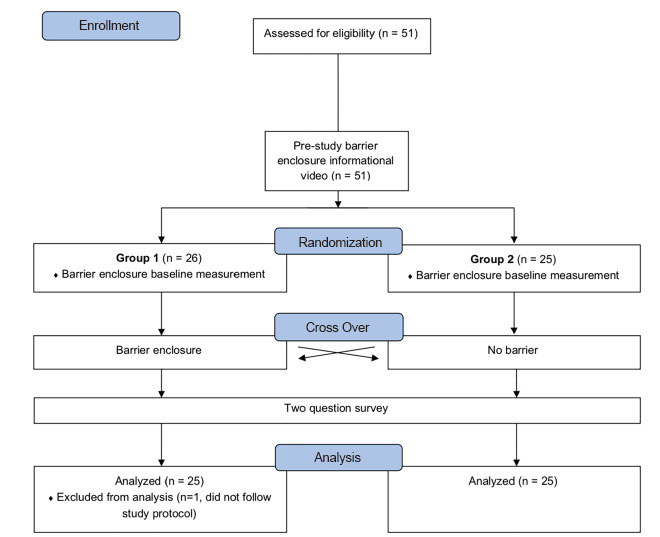
Design and flow of participants through the trial: All participants watched an introductory video and were then randomized into two groups. All groups performed a practice, barrier-enclosure baseline measurement, and depending on the group randomization, performed either a second trial with the barrier enclosure or no barrier enclosure. For the third trial the participants crossed over. All participants answered a two-question survey.

We conducted a nonblinded crossover simulation study involving the use of a video laryngoscope for simulated ETI under standard conditions with and without use of a barrier enclosure (Figure 1). Each participant watched an approximately four-minute video demonstrating proper use of the barrier enclosure and then had one intubation practice attempt with the barrier enclosure. Participants were assigned a number (consecutively) and randomized to either intubate with the barrier enclosure (odd numbers) or without (even numbers). They then crossed over and intubated without (odd numbers) or with the barrier enclosure (even numbers). After completion of the intubation attempts, each participant was asked to answer two brief questions. The study was reviewed and considered exempt by our institutional review board.

**Figure 2:**
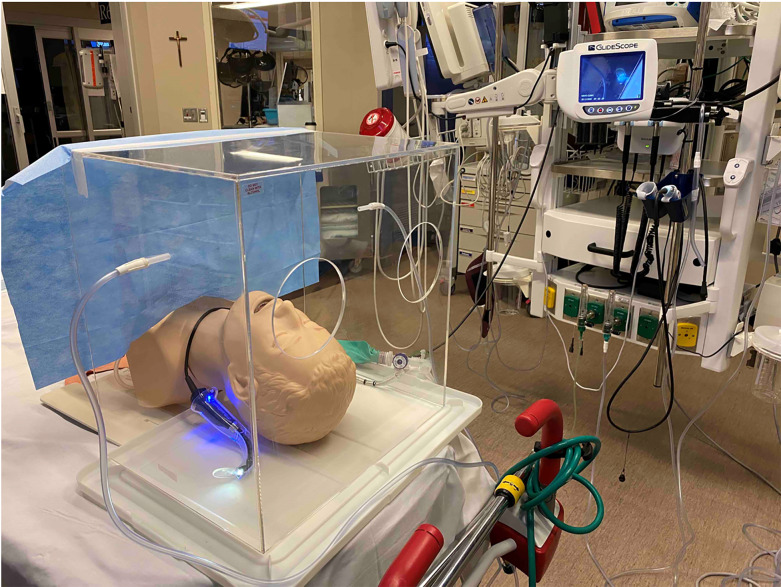
Set up with barrier enclosure placed around Airway Management Trainer (Laerdal Medical, Stavanger, Norway), GlideScope and endotracheal tube, as well as bag-valve mask within reach and visibility for participants, as well as the drape to protect the assistant. The barrier enclosure has a side port on each side, one for suction and one for oxygen insufflation to create a laminar flow and attempt to decrease droplet or aerosol spread through the circular cut outs or the draped side.

The barrier enclosure used was produced at our institution in collaboration with the Anatomic Modeling Lab and the Department of Engineering (Figure 2). It is modified from the box described by Hsien Yung Lai.^17^,^18^ It is constructed out of clear polycarbonate and measures 45.7 × 35.6 × 48.2 centimeters (cm) (18 × 14 × 19 inches). There are two circular cut-outs measuring 12.7 cm (5 inches) in diameter and placed 12.7 cm (5 inches) apart. The center of the holes are at a height of 30.5 cm (12 inches). The device additionally has side ports allowing oxygen inflow on one side and suction outflow on the opposite side. The idea is to create laminar flow in an attempt to capture small droplets or aerosols. The impact on aerosol and droplet capture, however, has not been confirmed. The enclosure is open on the side toward the patient’s feet and is covered with a disposable surgical drape to further mitigate droplet and aerosol spread and allow a second provider to pass tools to the proceduralist as needed.

The procedures were performed using a GlideScope video laryngoscope (Verathon Inc., Bothell, WA) with a size 3 cradle and a 7.0 millimeter endotracheal tube (ETT) with a GlideRite rigid stylet (Verathon Inc., Bothell, WA). The Airway Management Trainer (Laerdal Medical, Stavanger, Norway) mannequin was selected based on a balance of portability and realism. However, given the rigid plastic plate that secures the trainer, it was found to be higher off the bed than a sample live patient. We used a plastic storage container lid to support the enclosure (Figure 2) and better replicate the height of a sample patient, which also matched the SimMan 3G mannequin (Laerdal Medical, Stavanger, Norway) with approximately 25 cm from the highest point (chin) to the top of the barrier enclosure. Two study authors were present for each measurement. One recorded the time (FP) while the other (SMY, BJS, or TAL) performed the role of an assistant provider, assisting with tasks identically both with and without the barrier enclosure. The assistance was meant to replicate that which is generally provided during an ETI and included the following: handing the ETT to the proceduralist; assisting with removal of the stylet once requested or initiated by the proceduralist; inflating the ETT cuff once the tube was properly placed; and providing initial ventilation.

The study took place in the emergency department and operating rooms at a large, tertiary academic medical center in May 2020. The simulation procedures were done in situ. Centers for Disease Control and Prevention (CDC) social distancing recommendations at the time of the study limited numbers of individuals who could meet, making large gatherings such as conferences or in-person teaching sessions impossible. The brief time requirement and in situ clinical setting allowed participants to complete the study while working clinically.

### Selection of Participants

We recruited healthy volunteers who were employed at our hospital and are experienced clinicians who regularly perform ETI as part of their clinical practice. Participants were reached by e-mail and in person and given information about the study.

### Outcome Measures

The primary outcome measure was time from placement of the laryngoscope blade at the lips to first successful ventilation of the lungs. This time period was chosen as it represents a period when the patient is most at risk for hypoxia and the time is almost entirely dependent on the proceduralist. Secondary outcomes included recording complications, such as failed attempt at intubation or right mainstem intubation, and post-study questionnaire. After completion of the intubation attempts, each participant was asked:

1. “Did you feel that the intubation box made the procedure more challenging? YES/NO. If YES, what was most difficult about using the intubation box?”
2. “Would you consider using this device in clinical practice? YES/NO. If NO, why not?”

### Primary Data Analysis

The primary outcome was comparison of the time to intubation (time from laryngoscope at the lips until the first ventilation) between intubation with and without the barrier enclosure. The predetermined meaningful difference in intubation outcome between arms was a time difference of greater than 10 seconds or failed intubation. With a predetermined sample size of 50 participants, we anticipated 90% power to detect a difference that was one-half the size of the standard deviation. We also considered the subjective responses from the participants regarding whether or not the barrier enclosure made the procedure more difficult and whether or not they would consider using it in clinical practice.

Continuous features are summarized as medians and interquartile ranges (IQR). Categorical features are summarized as counts and percentages. Differences in intubation times between experimental conditions were assessed using paired-sample t-tests. The proportion of survey responses indicating “Yes” for each question was compared to a baseline 50% response rate using a one-proportion Z test. We computed confidence intervals (CI) for survey response rates using an asymptotic Gaussian approximation. All tests were two-sided and p-values below 0.05 were considered significant. For intubation times, a difference of greater than 10 seconds was predetermined as the threshold for clinical significance.

## RESULTS

### Characteristics of Study Subjects

In total, 51 participants took part in this study including 22 anesthesiologists and 29 emergency physicians, nurse practitioners, or physician assistants. One participant had multiple practice attempts and was excluded. Cohort demographics are given in Table 1. Data was available for all 50 included participants for primary analysis.

### Main Results

Table 2 provides a summary of intubation times for the practice, barrier enclosure, and no-barrier trials for each of the demographic sub-groups. Overall, time to intubation for the practice trial was the longest, with a median intubation time of 23.6 seconds (IQR: 18.8 – 28.9). Barrier enclosure trials were the second longest, with a median time of 20.5 seconds (IQR: 16.3 – 25.8), and no-barrier enclosure trials were the shortest with a median time of 16.7 (IQR: 10.8 – 19.1) seconds. There were three complications reported during the practice intubations: one right mainstem intubation and two episodes of the stylet being removed but then reinserted in order to appropriately advance the tube. No complications occurred during either the barrier enclosure trials or the no-barrier trials.

**Figure 3:**
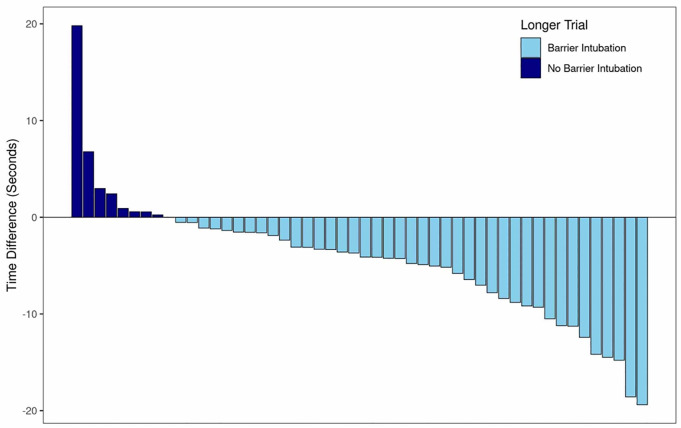
Difference in intubation time comparing use of barrier with use of no-barrier enclosure. Positive numbers reflect a longer time without a barrier enclosure; negative numbers reflect a longer time with the barrier enclosure.

Figure 3 shows the difference in intubation times for the barrier enclosure and no-barrier trials for all participants. Of the 50 participants, 42 (84%) took longer in the barrier enclosure intubation compared to the no-barrier trial. The barrier intubation was found to take significantly longer than the no-barrier intubation, with an average increased intubation time of 4.5 seconds (95% CI, 2.7–6.4, p < .001). Although the increase in time did not meet the predetermined overall clinical significance threshold of 10 seconds, in nine participants the intubation time was prolonged by more than 10 seconds for the barrier enclosure compared to no barrier. In addition, when comparing the practice and no-barrier trials, the practice trials were an average of 9.8 seconds longer (95% CI, 6.3 – 13.3, p < .001).

**Figure 4:**
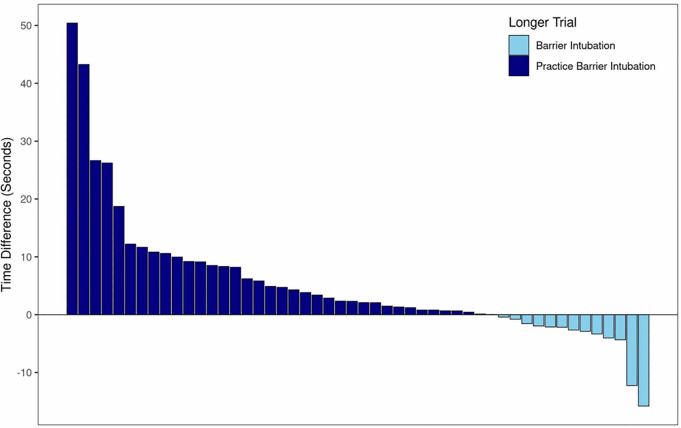
Difference in intubation time for practice and follow-up use of barrier enclosure. Positive numbers reflect a longer time during the practice barrier enclosure intubation (prior to study); negative numbers reflect a longer time during the follow-up barrier-enclosure intubation (during study).

Figure 4 shows the difference in intubation times for the practice barrier enclosure and follow-up barrier enclosure trials for all participants. Overall, the practice intubation took significantly longer than the follow-up barrier shield intubation, with an average increased intubation time of 5.2 seconds (95% CI, 2.0–8.5, p = .002). Out of the 50 participants, 13 (26%) took longer in the follow-up barrier intubation compared to the practice.

Table 3 provides details regarding the respondents who found the barrier enclosure made intubation more difficult and those who would consider using it in a clinical setting. Forty-eight percent (24/50) of participants indicated that the barrier enclosure made the intubation more challenging (p = 0.89). Reasons cited for increased difficulty included the following: challenges with the stylet removal; decreased dexterity and range of motion; trouble handling the ETT within the enclosure; and difficulty inserting the laryngoscope and ETT into the mouth. Ninety percent (45/50) of respondents indicated they would consider using a barrier enclosure in clinical practice (p<0.001). Twenty-one of the 24 (88%) participants who indicated that the barrier enclosure made the procedure more challenging would still consider using it in clinical practice.

## DISCUSSION

Protection of healthcare providers from COVID-19 infection while allowing safe care of patients is paramount. PPE supply shortages have been an ongoing dilemma during the pandemic. For these reasons, innovative strategies to decrease contagion during AGP are welcome. Furthermore, even if PPE supplies are robust, breaches in PPE during AGP and in donning and doffing can occur. Therefore, strategies to decrease droplet or aerosol spread of virus during airway management can be helpful in all settings. The barrier enclosure device may offer one such benefit. However, its safety has not previously been demonstrated.

The authors applaud Dr. Lai for allowing open access to the design and rationale of his novel “aerosol box.”^17^ We have modified our own barrier enclosure to allow additional space for tube passage, stylet removal, bag-valve mask ventilation, and even use of a bougie or other airway adjuncts. Our barrier enclosure uses tubed-in oxygen and high-flow suction to create laminar air flow within the enclosure. It seems unlikely that any barrier enclosure can eliminate aerosolization of viral particles; the term “aerosol box,” as pointed out by Chan, is somewhat of a misnomer.^22^ Therefore, use of a barrier enclosure does not preclude the need for full PPE. This tempers the potential benefits of these devices and must be weighed against the potential risks of their use.

In our study cohort, 48% of the participants felt using a barrier enclosure made intubation more challenging, yet 90% of the participants would still consider using it with a real patient. This highlights that the participants are willing to accept a more challenging and potentially longer intubation process to further minimize droplet and aerosol spread. While both video and direct laryngoscopy are regularly performed at our institution, in response to the COVID-19 pandemic, all emergent intubations are initially performed with video laryngoscopy. This is consistent with previously mentioned recommendations to maximize first-pass success.^14^ Video laryngoscopy has been shown to have higher first-pass success rates and fewer complications.^29^,^30^ In addition, direct laryngoscopy generally requires that the proceduralist’s face is closer to the patient’s mouth than is required for video laryngoscopy. For these reasons, we chose to test the barrier shield using the video laryngoscopy technique.

Our results confirmed that first-pass ETI with the video laryngoscopy technique by experienced clinicians was delayed by an average of 4.5 seconds when using a barrier enclosure. For most situations, this level of delay is likely of no significant consequence to the patient. However, the delay was seen in an uncomplicated simulated intubation and could be much greater when dealing with a difficult airway situation. We did see an expected improvement from the initial baseline use of the barrier enclosure to the second attempt with the device, decreasing the time to intubation by an average of 5.2 seconds. We do not know whether additional practice would further narrow the delay compared to intubation without a barrier enclosure, but our findings suggest that even one practice with the device was helpful.

## LIMITATIONS

We tested only a standard adult intubation using a video laryngoscope technique. We did not assess the impact the barrier enclosure would have on more challenging intubations or other techniques such as direct laryngoscopy or use of a gum elastic bougie. Neither the researchers nor the participants were blinded as to whether or not they were using the barrier enclosure. While the research team attempted to be consistent across groups, it is possible that the lack of blinding could have affected how assistance was given to participants. Also, the participants were aware they were being timed. While we encouraged them to try and perform the procedure as they would in an actual clinical setting, it is possible that some rushed to try and complete the procedure in a shorter period of time. The study was conducted in situ to be able to include as many participants while they were working clinically. In the interest of time, we were not able to conduct a second trial with the same participants to confirm our findings. We did record the year of each resident participant, but did not capture years of experience for attendings.

While delays in first-pass intubation success have been associated with worse patient outcomes,^26^–^28^ there is not a clear cut-point as to when delays become clinically meaningful. Based on our clinical experience, we chose a delay of more than 10 seconds as potentially clinically important during this phase of the procedure when the patient is paralyzed and at highest risk for hypoxia; however, there is little data to support any specific time point.

Many modifications have now been made to the originally described “aerosol box,” which may limit applicability if using a different type of barrier enclosure. Finally, this was a simulation study, which limits applicability to actual patients. While this was a simulated study and could not completely replicate actual clinical conditions, we did not feel it would be ethical to proceed with initial testing of this novel device on actual patients. However, simulation can be valuable in testing innovations in healthcare.^31^ Simulation has also been shown to be more effective than non-simulation techniques in teaching airway management,^32^ and mannequin-based models produce similar intubation times and first-pass success compared to cadaver models.^33^

## CONCLUSION

Whether or not to use a barrier enclosure is a decision that should be made carefully. Given the minimal increased time to first-pass success in an uncomplicated airway along with potential to decrease droplet spread during endotracheal intubation, use of a barrier enclosure appears to be an acceptable technique for those who are familiar with the device and the necessary adaptations to complete the procedure. Further research should focus on the impact of barrier enclosure use during difficult intubation scenarios and actual clinical encounters. Additionally, further robust investigation into how well these devices reduce droplet or aerosol spread of virus would also be of interest.

## Figures and Tables

**Table 1:** Summary of cohort demographics in trial of using a barrier enclosure box for intubation.

	Group 1: Box – No Box		Group 2: No Box - Box	

	Anesthesia (N = 10)	EM (N = 15)	Anesthesia (N = 11)	EM (N = 14)
Gender				
Male	9 (90%)	6 (40%)	6 (55%)	9 (64%)
Female	1 (10%)	9 (60%)	5 (45%)	5 (36%)
Role				
Attending	9 (90%)	6 (40%)	10 (91%)	8 (57%)
Nurse Practitioner/Physician Assistant	0 (0%)	2 (13%)	0 (0%)	2 (14%)
Resident	1 (10%)	7 (47%)	1 (9%)	4 (29%)
Year of Residency	1 PGY 4	2 PGY 1 4 PGY 2 1 PGY 3	1 PGY 3	3 PGY 1 1 PGY 2
Prior Experience with Barrier Enclosure				
Yes	2 (20%)	1 (7%)	2 (19%)	0 (0%)
No	8 (80%)	14 (93%)	9 (81%)	14 (100%)

*EM*, emergency medicine; *PGY*, postgraduate year.

**Table 2:** Summary of intubation time (seconds)

	Practice Median [IQR]	Barrier Median [IQR]	No Barrier Median [IQR]
Overall	23.6 [18.8 – 28.9]	20.5 [16.3 – 25.8]	16.7 [10.8 – 19.1]
Gender			
Male	21.0 [16.0– 24.6]	17.3 [13.2 – 23.5]	14.63 [10.1 – 18.5]
Female	27.3 [23.7 – 30.6]	22.4 [18.2 – 29.9]	17.5 [16.7 – 22.5]
Specialty			
EM	27.4 [23.3 – 34.0]	24.4 [20.7 – 29.7]	17.8 [16.7 – 20.7]
Anesthesiology	17.8 [13.3 – 23.6]	15.5 [12.3 – 17.2]	10.4 [8.6 – 15.4]
Role			
Attending	23.6 [16.9 – 28.5]	17.3 [13.8 – 22.6]	15.6 [10.1 – 19.2]
Nurse Practitioner/Physician Assistant	31.1 [28.5 – 32.8]	28.1 [25.5 – 34.8]	17.6 [16.9 – 21.4]
Resident	21.9 [20.1 – 29.8]	22.1 [19.1 – 25.6]	17.0 [16.5 – 18.8]
Prior Experience with Barrier Enclosure			
No	24.3 [19.2 – 29.8]	20.63 [16.7 – 26.7]	16.7 [12.2 – 19.4]
Yes	18.7 [17.8 – 20.9]	15.46 [12.5 – 18.7]	10.6 [7.7 – 17.0]

*IQR*, interquartile range; *EM*, emergency medicine

**Table 3:** Summary of “yes” responses to survey questions.

	Respondents	Q1: More Challenging?	Q2: Use in Practice?
Overall	50	24 (48%)	45 (90%)
Gender			
Male	30	13 (43%)	27 (90%)
Female	20	11 (55%)	18 (90%)
Specialty			
EM	29	19 (66%)	26 (90%)
Anesthesiology	19	5 (24%)	19 (91%)
Role			
Attending	33	13 (39%)	29 (88%)
Nurse Practitioner/Physician Assistant	4	2 (50%)	4 (100%)
Resident	13	9 (69%)	12 (92%)
Experience with Barrier Enclosure			
No	45	21 (47%)	40 (89%)
Yes	5	3 (60%)	5 (100%)

## References

[1] Lu H, Stratton CW, Tang YW (2020). Outbreak of pneumonia of unknown etiology in Wuhan, China: the mystery and the miracle. J Med Virol.

[2] Kakodkar P, Kaka N, Baig MN (2020). A comprehensive literature review on the clinical presentation, and management of the pandemic coronavirus disease 2019 (COVID-19). Cureus.

[3] Sohrabi C, Alsafi Z, O’Neill N (2020). World Health Organization declares global emergency: a review of the 2019 novel coronavirus (COVID-19). Int J Surg.

[4] WHO (2020). Coronavirus Disease (COVID-2019) Situation Report - 114.

[5] Mahase E (2020). Covid-19: WHO declares pandemic because of “alarming levels” of spread, severity, and inaction. BMJ.

[6] Pan A, Liu L, Wang C (2020). Association of public health interventions with the epidemiology of the COVID-19 outbreak in Wuhan, China. JAMA.

[7] Seto WH (2015). Airborne transmission and precautions: facts and myths. J Hosp Infect.

[8] Chan MTV, Chow BK, Lo T (2018). Exhaled air dispersion during bag-mask ventilation and sputum suctioning: implications for infection control. Sci Rep.

[9] Cook TM (2020). Personal protective equipment during the coronavirus disease (COVID) 2019 pandemic: a narrative review. Anaesthesia.

[10] van Doremalen N, Bushmaker T, Morris DH (2020). Aerosol and surface stability of SARS-CoV-2 as compared with SARS-CoV-1. N Engl J Med.

[11] Wilson NM, Norton A, Young FP (2020). Airborne transmission of severe acute respiratory syndrome coronavirus-2 to healthcare workers: a narrative review. Anaesthesia.

[12] Shanafelt T, Ripp J, Trockel M (2020). Understanding and addressing sources of anxiety among health care professionals during the COVID-19 pandemic. JAMA.

[13] CDC COVID-19 Response Team Morbidity & Mortality Weekly Report (2020). Prevention CfDCa. Characteristics of Health Care Personnel with COVID-19 — United States, February 12–April 9, 2020.

[14] Cook TM, El-Boghdadly K, McGuire B (2020). Consensus guidelines for managing the airway in patients with COVID-19: Guidelines from the Difficult Airway Society, the Association of Anaesthetists the Intensive Care Society, the Faculty of Intensive Care Medicine and the Royal College of Anaesthetists. Anaesthesia.

[15] Artenstein AW (2020). In pursuit of PPE. N Engl J Med.

[16] Lai YY, Chang CM (2020). [Ahead of Print]. A carton-made protective shield for suspicious/confirmed COVID-19 intubation and extubation during surgery. Anesth Analg.

[17] Lai HY (2020). Aerosol Box.

[18] Malik J, Jenner C, Ward P (2020). Maximising application of the aerosol box in protecting healthcare workers during the COVID-19 pandemic. Anaesthesia.

[19] Begley JL, Lavery KE, Nickson CP (2020). The aerosol box for intubation in COVID-19 patients: an in-situ simulation crossover study. Anaesthesia.

[20] Administration USFaD (2020). Emergency Use Authorization for protective barrier enclosures.

[21] Canelli R, Connor CW, Gonzalez M (2020). Barrier enclosure during endotracheal intubation. N Engl J Med.

[22] Chan A (2020). Should we use an “aerosol box” for intubation?.

[23] Kearsley R (2020). Intubation boxes for managing the airway in patients with COVID-19. Anaesthesia.

[24] Aberle SJ, Sandefur BJ, Sunga KL (2015). Intubation efficiency and perceived ease of use of video laryngoscopy vs direct laryngoscopy while wearing HazMat PPE: a preliminary high-fidelity mannequin study. Prehosp Disaster Med.

[25] Adler MD, Krug S, Eiger C (2020). [Ahead of Print]. Impact of personal protective equipment on the performance of emergency pediatric tasks. Pediatr Emerg Care.

[26] Sakles JC, Chiu S, Mosier J (2013). The importance of first-pass success when performing orotracheal intubation in the emergency department. Acad Emerg Med.

[27] Mort TC (2004). Emergency tracheal intubation: complications associated with repeated laryngoscopic attempts. Anesth Analg.

[28] Natt BS, Malo J, Hypes CD (2016). Strategies to improve first attempt success at intubation in critically ill patients. Br J Anaesth.

[29] Liu DX, Ye Y, Zhu YH (2019). Intubation of non-difficult airways using video laryngoscope versus direct laryngoscope: a randomized, parallel-group study. BMC Anesthesiol.

[30] De Jong A, Molinari N, Conseil M (2014). Video laryngoscopy versus direct laryngoscopy for orotracheal intubation in the intensive care unit: a systematic review and meta-analysis. Intensive Care Med.

[31] Madani A, Gallix B, Pugh CM (2017). Evaluating the role of simulation in healthcare innovation: recommendations of the Simnovate Medical Technologies Domain Group. BMJ STEL.

[32] Kennedy CC, Cannon EK, Warner DO (2014). Advanced airway management simulation training in medical education: a systematic review and meta-analysis. Crit Care Med.

[33] Pedigo R, Tolles J, Watcha D (2019). Teaching endotracheal intubation using a cadaver versus a manikin-based model: a randomized controlled trial. West J Emerg Med.

